# Association between vitamin D and serum uric acid in a large Chinese cohort of middle-aged and elderly Chinese men and women

**DOI:** 10.3389/fnut.2026.1726451

**Published:** 2026-02-20

**Authors:** Rong Cao, Bingchao Xu, Yubao Wang, Xinzheng Lu, Xianhui Qin, Xiaoshu Cheng, Guifan Sun, Jingang Yang, Ningling Sun, Gang Sun, Hui Shi, Han-Ping Shi, Liangdi Xie

**Affiliations:** 1 Department of Geriatrics, The First Affiliated Hospital of Fujian Medical University, Fuzhou, Fujian, China; 2 Department of General Medicine, The First Affiliated Hospital of Fujian Medical University, Fuzhou, Fujian, China; 3 Clinical Research Center for Geriatric Hypertension Disease of Fujian Province, The First Affiliated Hospital of Fujian Medical University, Fuzhou, China; 4 Branch of National Clinical Research Center for Aging and Medicine, The First Affiliated Hospital of Fujian Medical University, Fuzhou, China; 5 Department of Geriatrics, Binhai Campus of the First Affiliated Hospital, National Regional Medical Center, Fujian Medical University, Fuzhou, China; 6 Department of Neurosurgery, Lianyungang First People's Hospital, Jiangsu, China; 7 Department of Cardiology, Lianyungang Municipal Oriental Hospital, Jiangsu, China; 8 Department of Cardiology, First Affiliated Hospital, Nanjing Medical University, Nanjing, China; 9 Division of Nephrology, Nanfang Hospital, Southern Medical University, Guangzhou, China; 10 National Clinical Research Center for Kidney Disease, Guangzhou, China; 11 Department of Cardiology, The Second Affiliated Hospital of Nanchang University, Nanchang, China; 12 Research Center of Environmental and Non-Communicable Disease, School of Public Health, China Medical University, Shenyang, China; 13 Coronary Heart Disease Center, Department of Cardiology, Fuwai Hospital, National Center for Cardiovascular Diseases, Chinese Academy of Medical Science and Peking Union Medical College, Beijing, China; 14 Department of Hypertension at Heart Center, Peking University People's Hospital, Beijing, China; 15 Research Institute of Hypertension, Department of Cardiovascular Medicine, The Second Affiliated Hospital of Baotou Medical College, Baotou, Inner Mongolia, China; 16 Department of Gastrointestinal Surgery, Beijing Shijitan Hospital, Capital Medical University, Beijing, China; 17 Department of Clinical Nutrition, Beijing Shijitan Hospital, Capital Medical University, Beijing, China; 18 Key Laboratory of Cancer FSMP for State Market Regulation, Beijing, China

**Keywords:** Chinese cohort, hyperuricemia, serum uric acid, vitamin D, vitamin D deficiency

## Abstract

**Background:**

Vitamin D is essential for calcium homeostasis, bone health, and immune function, yet its association with serum uric acid (UA) remains uncertain. This study evaluates vitamin D status in Chinese adults and explores its sex- and age-specific relationships with UA and hyperuricemia (HUA).

**Methods:**

We conducted a cross-sectional analysis of 15,116 males and 25,895 females from The China Precision Nutrition and Health KAP Real World Study (CPNAS). Restricted cubic spline (RCS) visualized dose-response relationships, while multivariate regression assessed associations between vitamin D and UA/HUA. Subgroup analyses (age < 70 vs. ≥70, male vs. female) explored potential variations.

**Results:**

The study revealed a high prevalence of vitamin D deficiency (33.5%) and insufficiency (53%) in Chinese adults. Among males under 70 years, we observed an inverse J-shaped relationship between vitamin D and serum UA levels (*P*-non-linear = 0.046). Compared to those with vitamin D < 22 ng/ml, participants with moderate levels (22–30 ng/ml) showed significantly lower UA (β = −5.40 to −4.36, all *P* < 0.05), while no significant reduction occurred at higher concentrations (≥25.8–30 ng/ml, *P* > 0.05). Notably, this association was absent in males ≥70 years. In contrast, females exhibited a consistent positive linear relationship between vitamin D and UA. These patterns were similarly observed for hyperuricemia risk in both sexes.

**Conclusion:**

Vitamin D levels are differentially associated with UA and HUA based on sex and age, highlighting the need for personalized approaches in managing vitamin D and UA metabolism.

**Clinical trial registration:**

This trial was registered at https://www.chictr.org.cn/hvshowproject.html?id=178499&v=2.2, as ChiCTR2100051983.

## 1 Introduction

Uric acid (UA), the final metabolite of purine metabolism in humans, is commonly considered a metabolic waste product. However, numerous investigations have demonstrated that UA plays two distinct roles in oxidative stress: it can act as a pro-oxidant, causing damage through crystallization, as well as an anti-oxidant, providing protective effects (1). It is becoming increasingly clear that UA is not a biologically inert substance, but it has multiple biological functions. Serum UA levels are determined by the balance between its production and excretion. Any imbalances in this process can lead to abnormal UA levels, resulting in dysuricemia, which includs both hyperuricemia (HUA) and hypouricemia. HUA has been a major focus of research and concern in the past, as it can lead to clinical symptoms such as gout. In contrast, hypouricemia has long been neglected. Initially hypouricemia was regarded as a biochemical disorder with no clinical significance. However, recent studies of UA have revealed new insights, showed that hypouricemia is a pathological condition that increases the risk of several diseases, including chronic kidney disease (CKD) (2), dementia (3), and Parkinson disease (4). Therefore, it is crucial to acknowledge that both elevated and decreased UA levels can have adverse effects on the human body.

The role of vitamin D in regulating skeletal and mineral ion homeostasis is well established (5). Additionally, the presence of vitamin D receptor and vitamin D-metabolizing enzymes in nearly all human tissues suggests that vitamin D plays a widespread role in overall human health. Accumulating evidence also indicates that low vitamin D levels are associated with an increased risk of various common disorders, including cardiovascular disease (CVD) (6), malignant (7), type 2 diabetes mellitus (T2DM) (8), autoimmune diseases (9), high blood pressure, depression, and overall mortality (10). However, excessive vitamin D concentrations (>100 ng/ml), also known as vitamin D toxicity, can be detrimental. It may lead to hypercalciuria, which can result in acute kidney disease (AKD) and vascular calcification (11).

The need for data on the representational status and intake of vitamin D has been a focus of research for over a decade. Epidemiological studies have documented that the global prevalence of vitamin D levels < 25/30 and < 50 nmol/L ranges from approximately 5 to 18% and 24 to 49%, respectively (12). Notably, considering factors such as latitude, genetics, lifestyle, body composition, and dietary intake, the current vitamin D status in China remains unclear and requires further clarification. Given the crucial role of vitamin D and the importance of maintaining appropriate levels of UA for human health, the potential contribution of vitamin D to serum UA concentrations remains uncertain and warrants further investigation. The association between vitamin D and UA levels remains elusive in existing studies. Some studies have suggested that there is no relationship between vitamin D and serum UA (13, 14), however, other studies have indicated a link between vitamin D and UA levels, but the evidence is less clear and controversial. For example, some studies have found a positive linear correlation between vitamin D and serum UA (15–17), while other studies have found a negative correlation between vitamin D and HUA (18, 19), and several studies have found a non-linear relationship between vitamin D and serum UA/HUA (20, 21). Although none of these studies reported a sex and age-specific association between vitamin D and serum UA in detail. Thus, an updated relationship between vitamin D and UA among elder Chinese is critically needed. The primary objective of this study was to depict the association between vitamin D and UA. As serum UA levels are vary between the sexes (17) and vitamin D levels are vary in different ages (22), it is therefore important to take gender and age differences into account when exploring the relationship between vitamin D and UA, to provide clues and evidence for the application of precision nutrition in China (23, 24). We hypothesized that plasma 25(OH)D is associated with serum UA in Chinese adults and that this association differs significantly by sex and age.

## 2 Methods

### 2.1 Participants

The subjects were over 18 years old in Rongcheng, China, who participated in The China Precision Nutrition and Health KAP Real World Study (CPNAS; registration number: ChiCTR2100051983). CPNAS is a long-term, multi-center, prospective, observational, real-world study conducted in different regions of China. Detailed descriptions of CPNAS have been outlined in earlier publications. Each eligible participant achieved informed consent (25, 26).

A total of 41,011 participants from the CPNAS were enrolled in our study. Participants were excluded based on the following criteria: (1) taking Enalapril folic acid (*n* = 9,031); (2) taking vitamin D (*n* = 1,819); (3) taking multivitamins (*n* = 500); (4) taking multivitamins minerals (*n* = 220); and (5) missing data (on vitamin D, uric acid, or covariates) or having vitamin D or UA values outside 3 standard deviations (SD; *n* = 1,114).

### 2.2 Data collection

Age, sex, body mass index (BMI), medical history, personal history (smoking, drinking, hypertension, diabetes mellitus, and hyperlipidemia), vitamin D, UA, creatinine (CREA), glucose (GLU), triglyceride (TG), total cholesterol (TC), low-density lipoprotein cholesterol (LDL-C), systolic blood pressure (SBP), diastolic blood pressure (DBP), and heart rate (HR) of all participants were extracted from the CPNAS.

The measurement of vitamin D was performed using Liquid Chromatography-Tandem Mass Spectrometry (LC-MS/MS). Detailed methods for the LC-MS/MS measurements can be found in a previous publication (27). Vitamin D deficiency was defined as a vitamin D < 20 ng/ml, insufficiency as 20–30 ng/ml, and sufficiency as a vitamin D >30 ng/ml (28). HUA was defined as serum UA ≥420 μmol/L regardless of males or females (29). Hypertension (HTN) is diagnosed with any of the following: (1) previously diagnosed HTN; (2) taking hypertensive medication; (3) being informed by a licensed physician of hypertension or stated in the questionnaire to take prescribed medication for hypertension; and (4) measuring the participant's average SBP ≥140 mmHg and/(or) DBP ≥90 mmHg (30). Diabetes mellitus (DM) was diagnosed in subjects who met any of the following criteria: (1) previously diagnosed DM; (2) GLU ≥7.0 mmol/L; and (3) use of diabetes medication or insulin (31). The diagnostic criteria for hyperlipidemia are any of the following: (1) TC ≥6.19 mmol/L; (2) TG ≥2.3 mmol/L; (3) LDL-C ≥4.1 mmol/L; (4) previously diagnosed hyperlipidemia; and (5) use of lipid-lowering medication (32).

### 2.3 Statistical analysis

Normally distributed variables were expressed as mean ± standard deviation (SD), and abnormally distributed variables were shown as the median (interquartile range, IQR). Categorical data were expressed as numbers and percentage (*n*, %).

Restricted cubic spline (RCS) was used to visualize the dose-response relationship between vitamin D and serum UA. Subsequently, multivariate regression analysis was performed with UA/HUA as the dependent variable and vitamin D as the main independent variable. The vitamin D levels were modeled in quartiles (Q1, Q2, Q3, and Q4) categories (Q1, Q2-Q3, and Q4) and clinical cut-off values (< 20, 20–30, >30 ng/ml). Both the non-adjusted and multivariate-adjusted models were used. Model 1 was the crude model with no adjustments. Model 2 was adjusted for age, sex, BMI, smoking status, drinking status, HTN, DM, hyperlipidemia, and CREA. Model assumptions and fit were verified using residual plots, goodness-of-fit tests, and multicollinearity assessments (e.g., variance inflation factor, VIF; generalized variance inflation factor, GVIF). Finally, *post hoc* subgroup analyses (age < 70 vs. ≥70, male vs. female) were performed.

To verify the robustness of our findings, we performed sensitivity analyses by: (1) excluding participants with CKD; and (2) including participants taking vitamin D supplements (who were excluded from the primary analysis) to assess the potential influence of exogenous vitamin D intake. All *P*-values were 2-tailed. A *P*-value < 0.05 was considered statistically significant. All of the statistical analyses were performed with R statistical software, V1.7 (https://www.r-project.org).

## 3 Results

### 3.1 Baseline characteristics

**Figure 1:**
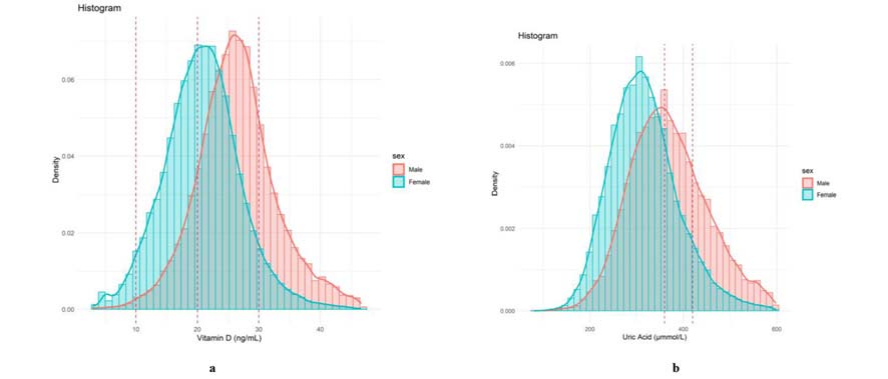
Distribution of vitamin D and serum UA by sex. **(a)** Distribution of vitamin D by sex; **(b)** distribution of serum UA by sex.

A total of 41,011 participants were selected for the final data analysis, with a median age of 65.4 (58.1, 71.5) years, of which 63.1% were female (Table 1). As shown in Figure 1, males had higher levels of both vitamin D and serum UA compared to females. Among all participants, 33.5% had vitamin D deficiency, 53% had insufficiency, and 13.5% had sufficient vitamin D levels. In males, the prevalence of vitamin D deficiency, insufficiency, and sufficiency was 14.0, 60.8, and 25.2%, respectively. In females, these values were 44.8, 48.5, and 6.7%, respectively. The overall prevalence of HUA in our study was 18.4%, with 27.7% of man and 13% of women affected. Compared to female, male had higher vitamin D (26.2 vs. 20.8, *P* < 0.001) and serum UA (363.0 vs. 311.0, *P* < 0.001) levels. Furthermore, compared to females, males tended to be older, with lower BMI, TG, TC, LDL-C, SBP, DBP and HR, and with higher CREA, had a higher proportion of current smoking and drinking, and lower proportion of individuals with HTN, DM, and hyperlipidemia.

**Table 1:** Baseline characteristics of participants by the sex.

**Variables**	**Total (*n* = 41,011)**	**Male (*n* = 15,116)**	**Female (*n* = 25,895)**	***P***
Age (years)^a^	65.4 (58.1, 71.5)	67.3 (59.8, 73.1)	64.0 (57.4, 70.3)	< 0.001
BMI (kg/m^2^)^a^	25.6 (23.2, 28.0)	24.9 (22.6, 27.3)	25.9 (23.6, 28.5)	< 0.001
**Smoking**, ***n*** **(%)**^b^				< 0.001
Current	6,716 (16.40)	6,542 (43.30)	174 (0.70)	
Quit	2,889 (7.00)	2,848 (18.80)	41 (0.20)	
Never smoked or otherwise	31,377 (76.60)	5,722 (37.90)	25,655 (99.20)	
**Drinking**, ***n*** **(%)**^b^				< 0.001
Never	30,419 (74.40)	5,960 (39.50)	24,459 (94.7)	
Current	9,232 (22.60)	7,974 (52.80)	1,258 (4.90)	
Abstinence or others	1,260 (3.10)	1,155 (7.70)	105 (0.40)	
**HTN**, ***n*** **(%)**^b^				0.026
No	19,158 (46.9)	7,179 (47.6)	11,979 (46.5)	
Yes	21,716 (53.1)	7,906 (52.4)	13,810 (53.5)	
**DM**, ***n*** **(%)**^b^				< 0.001
No	34,098 (84.0)	12,705 (84.9)	21,393 (83.5)	
Yes	6,484 (16.0)	2,259 (15.1)	4,225 (16.5)	
**Hyperlipidemia**, ***n*** **(%)**^b^				< 0.001
No	23,450 (57.3)	10,283 (68.1)	13,167 (51.0)	
Yes	17,461 (42.7)	4,813 (31.9)	12,648 (49.0)	
**HUA**, ***n*** **(%)**^b^				< 0.001
No	33,387 (81.6)	10,917 (72.3)	22,470 (87.0)	
Yes	7,548 (18.4)	4,184 (27.7)	3,364 (13.0)	
CREA (μmol/L)^a^	63.0 (56.0, 72.0)	71.0 (64.0, 80.0)	59.0 (53.0, 65.0)	< 0.001
SBP (mmHg)^a^	137.7 (125.0, 151.7)	136.7 (124.7, 149.7)	138.7 (125.3, 152.3)	< 0.001
DBP (mmHg)^a^	81.0 (74.3, 88.0)	82.3 (75.3, 89.7)	80.3 (73.7, 87.3)	< 0.001
HR (beats/min)^a^	73.3 (66.7, 81.3)	72.0 (64.7, 80.3)	74.0 (67.7, 81.7)	< 0.001
GLU (mmol/L)^a^	5.40 (5.00, 6.00)	5.40 (5.00, 6.00)	5.40 (5.00, 6.00)	0.923
TG (mmol/L)^a^	1.40 (1.00, 2.10)	1.20 (0.90, 1.80)	1.60 (1.10, 2.20)	< 0.001
TC (mmol/L)^a^	5.50 (4.80, 6.20)	5.20 (4.60, 5.90)	5.60 (4.90, 6.40)	< 0.001
LDL-C (mmol/L)^a^	3.20 (2.70, 3.70)	3.00 (2.60, 3.50)	3.30 (2.80, 3.90)	< 0.001
UA (μmol/L)^a^	328.0 (279.0, 385.0)	363.0 (310.0, 422.0)	311.0 (266.0, 359.0)	< 0.001
Vitamin D (ng/ml)^a^	22.7 (18.4, 27.0)	26.2 (22.4, 30.1)	20.8 (16.8, 24.6)	< 0.001
**Vitamin D status**, ***n*** **(%)**^b^				< 0.001
Deficiency	13,720 (33.5)	2,115 (14.0)	11,605 (44.8)	
Insufficiency	21,751 (53.0)	9,188 (60.8)	12,563 (48.5)	
Sufficiency	5,540 (13.5)	3,813 (25.2)	1,727 (6.7)	

BMI, body mass index; UA, uric acid; CREA, creatinine; GLU, glucose; TG, triglyceride; TC, total cholesterol, LDL-C, low density lipoprotein cholesterol; SBP, systolic blood pressure; DBP, diastolic blood pressure; HR, heart rate; HTN, hypertension; DM, diabetes mellitus; HUA, hyperuricemia.

^a^Data was presented as median (interquartile range, IQR).

^b^Data was presented as n (%).

### 3.2 Association between vitamin D and serum UA

**Figure 2:**
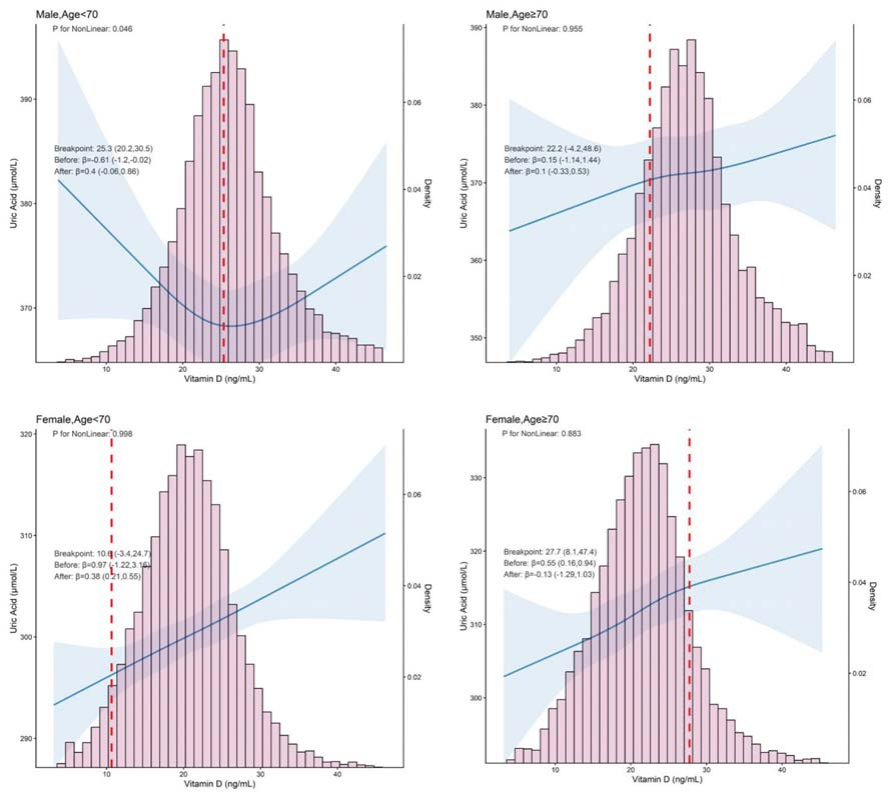
Restricted cubic spline analysis of the association between serum vitamin D and UA levels, stratified by sex and age (< 70 vs. ≥70 years). Adjusted age, sex, BMI, smoking, drinking, CREA, hyperlipidemia, HTN, and DM. BMI, body mass index; UA, uric acid; CREA, creatinine; HTN, hypertension; DM, diabetes mellitus.

The RCS revealed an inverse J-shaped, non-linear dose-response relationship between vitamin D and serum UA in males, particularly in those younger than 70 years, This relationship was observed in both the non-adjusted (*P*-non-linear = 0.008) and an adjusted model (*P*-non-linear = 0.046). However, a positive linear relationship between vitamin D and serum UA in other groups (males and age ≥70 and females) in an adjusted model (Figure 2). The multivariate linear regression results for the association between vitamin D and serum UA are shown in Table 2 and Supplementary Table S3. Diagnostic plots and tests indicated that the regression models met the assumptions of linearity, normality of residuals, and homoscedasticity, with no evidence of significant multicollinearity (VIF, GVIF < 2; Supplementary Table S5). In males < 70 years, after adjusting all confounding factors, serum UA levels decreased with increasing vitamin D levels across different vitamin D categories and clinical cut-off values. Specifically, for participants with vitamin D levels between 22–25.8, 22–29.6, and 20–30 ng/ml, serum UA levels decreased as vitamin D increased (β = −5.40, 95% CI = −9.82, −0.99; *P* = 0.017; β = −4.36, 95% CI = −8.18, *P* = 0.026; β = −4.65, 95% CI = −9.14, −0.16, *P* = 0.042). The associations remained statistically significant after FDR correction. However, when vitamin D levels were ≥25.8, 29.6, and 30 ng/ml, no significant association was found between vitamin D and serum UA (*P* > 0.05 for all). In females, a significant positive linear relationship between vitamin D and serum UA was observed, regardless of whether vitamin D were treated as categorical variables (*P* all < 0.05) or as a continuous variable (per 10 ng/ml increment: β = 3.90, 95% CI: 2.30–5.40, *P* < 0.001). A similar trend was observed in the general population, where a significant positive correlation between vitamin D and serum UA was found. For the continuous variable, the relationship was also significant (per 10 ng/ml increment: β = 7.20, 95% CI: 6.10–8.20; *P* < 0.05 for all; Supplementary Table S1). Notably, the results remained consistent in sensitivity analyses after excluding participants with CKD or when including participants taking vitamin D supplements (Supplementary Tables S6–S23).

**Table 2:** Association between vitamin D and serum UA.

**Sex**	**Vitamin D**					**P**		**Vitamin D**			**P**	
**Female (*****n*** = **126,319)**	**Vitamin D (per 10 ng/ml increase) (*****n*** = **25,895)**	**Q1 (**<**16.8 ng/ml) (*****n*** = **6,467)**	**Q2 (16.8–20.8 ng/ml) (*****n*** = **6,475)**	**Q3 (20.8–24.6 ng/ml) (*****n*** = **6,479)**	**Q4 (**≥**24.6 ng/ml) (*****n*** = **6,474)**	***P*** **for trend**	***P*** **for FDR**	<**20 ng/ml (*****n*** = **11,605)**	**20–30 ng/ml (*****n*** = **12,563)**	≥**30 ng/ml (*****n*** = **1,727)**	***P*** **for trend**	***P*** **for FDR**
M1 [β (95% CI)]	4.18 (2.74, 5.62)	0 (Ref)	2.98 (0.45, 5.51)	5.02 (2.49, 7.55)	6.41 (3.88, 8.94)	< 0.001	0.004	Ref	3.31 (1.45, 5.16)	6.69 (2.98, 10.40)	< 0.001	0.004
M2 [β (95% CI)]	4.06 (.2.72, 5.41)	0 (Ref)	2.66 (0.32, 5.01)	4.16 (1.82, 6.50)	6.32 (3.97, 8.67)	< 0.001	0.004	Ref	3.27 (1.56, 4.99)	6.71 (3.24, 10.18)	< 0.001	0.004
**Male (*****n*** = **15,970)**	**Vitamin D (per 10 ng/ml increase) (*****n*** = **15,116)**	**Q1 (**<**22.4) (*****n*** = **3,779)**	**Q2 (22.4–26.2) (*****n*** = **3,779)**	**Q3 (26.2–30.1) (*****n*** = **3,778)**	**Q4 (**≥**30.1) (*****n*** = **3,780)**			<**20 (*****n*** = **2,115)**	**20–30 (*****n*** = **9,188)**	≥**30 (*****n*** = **3,813)**		
M1 [β (95% CI)]	0.60 (−1.43, 2.64)	Ref	−1.73 (−5.46, 1.99)	−2.13 (−5.86, 1.59)	0.22 (−3.51, 3.94)	0.967	0.967	Ref	−2.00 (−5.91, 1.90)	−0.12 (−4.51, 4.26)	0.793	0.793
M2 [β (95% CI)]	0.04 (−1.83, 1.92)	Ref	−1.57 (−4.99, 1.85)	−1.75 (−5.17, 1.67)	−0.39 (−3.82, 3.05)	0.807	0.807	Ref	−2.29 (−5.89, 1.30)	−1.22 (−5.28, 2.83)	0.759	0.759
**Male**<**70 (*****n*** = **9,368)**	**Vitamin D (per 10 ng/ml increase) (*****n*** = **9,368)**	**Q1 (**<**22) (*****n*** = **2,342)**	**Q2 (22–25.8) (*****n*** = **2,333)**	**Q3 (25.8–29.6) (*****n*** = **2,350)**	**Q4 (**≥**29.6) (*****n*** = **2,343)**			<**20 (*****n*** = **1,416)**	**20–30 (*****n*** = **5,756)**	≥**30 (*****n*** = **2,196)**		
M1 [β (95% CI)]	−0.27 (−2.8, 2.3)	Ref	−5.61 (−10.35, −0.86)	−4.21 (−8.94, 0.53)	−1.75 (−6.49, 2.99)	0.613	0.817	Ref	−4.21 (−9.02, 0.60)	−1.65 (−7.17, 3.88)	0.793	0.793
M2 [β (95% CI)]	−0.50 (–.2.9, 1.9)	Ref	−5.40 (−9.82, −0.99)	−3.31 (−7.73, 1.10)	−1.31 (−5.73, 3.11)	0.798	0.807	Ref	−4.65 (−9.14, −0.16)	−2.11 (−7.27, 3.06)	0.664	0.759
**Male** ≥**70 (*****n*** = **5,748)**	**Vitamin D (per 10 ng/ml increase) (*****n*** = **5,748)**	**Q1 (**<**23) (*****n*** = **1,437)**	**Q2 (23–26.8) (*****n*** = **1,437)**	**Q3 (26.8–30.6) (*****n*** = **1,437)**	**Q4 (**≥**30.6) (*****n*** = **1,437)**			<**20 (*****n*** = **699)**	**20–30 (*****n*** = **3,432)**	≥**30 (*****n*** = **1,617)**		
M1 [β (95% CI)]	3.71 (0.41, 7.01)	Ref	−2.09 (−8.08, 3.89)	3.92 (−2.06, 9.91)	5.55 (−0.44, 11.53)	0.019	0.038	Ref	3.84 (−2.82, 10.50)	6.03 (−1.23, 13.30)	0.110	0.220
M2 [β (95% CI)]	2.28 (−0.69, 5.25)	Ref	−0.25 (−5.60, 5.11)	2.96 (−2.39, 8.32)	3.33 (−2.05, 8.71)	0.129	0.258	Ref	4.33 (−1.66, 10.31)	3.94 (−2.60, 10.49)	0.387	0.759

M1: adjusted none. M2: adjusted age, sex, BMI, smoking, drinking, CREA, hyperlipidemia, HTN, and DM.

BMI, body mass index; UA, uric acid; CREA, creatinine; HTN, hypertension; DM, diabetes mellitus.

### 3.3 Association between vitamin D and HUA

**Figure 3:**
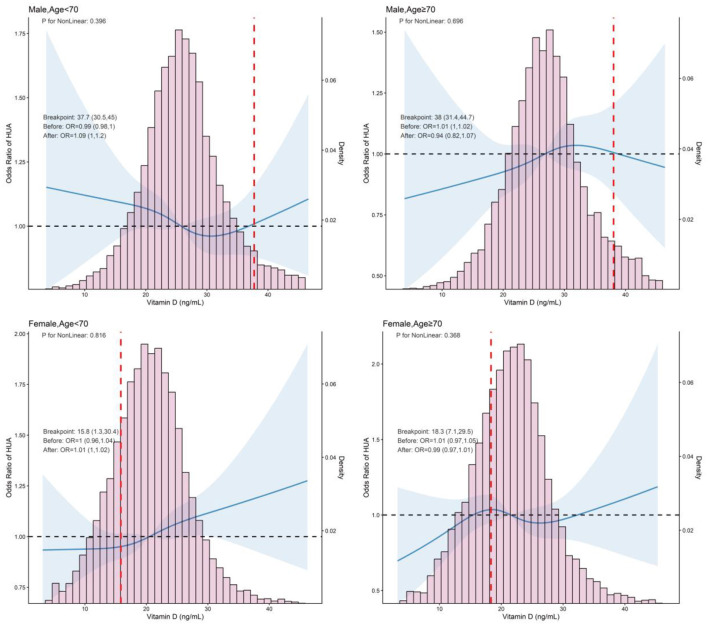
Restricted cubic spline analysis of the association between serum vitamin D and HUA, stratified by sex and age (< 70 vs. ≥70 years). Adjusted age, sex, BMI, smoking, drinking, CREA, hyperlipidemia, HTN, and DM. BMI, body mass index; UA, uric acid; CREA, creatinine; HTN, hypertension; DM, diabetes mellitus; HUA, hyperuricemia.

RCS between vitamin D and HUA revealed that there was an inverse J-shaped trend in the general population (*P* for non-linearity = 0.015), which was significant only in Model 1. In contrast, vitamin D and HUA were positively correlated linearly in both males and females (Figure 3). To further investigate the association between vitamin D and HUA, we performed a logistic regression analysis the results of which are presented in Table 3 and Supplementary Table S4. Model diagnostics indicated no significant multicollinearity among covariates (VIF, GVIF < 2) and adequate goodness-of-fit (Supplementary Table S5). When vitamin D was analyzed as a continuous variable, a significant positive correlation was found between vitamin D and HUA in both females and the general population (female, OR from 1.00 to 1.01, *P* < 0.05; general, OR from 1.01 to 1.01, *P* < 0.001). However, no significant correlation was observed between vitamin D and HUA in males. When vitamin D was analyzed as a categorical variable (quartile, three categories, or clinical cut-off values), a positive correlation between vitamin D levels and HUA was still present in the general population (*P* < 0.05), In contrast, no significant association was found between vitamin D and HUA in either males or females. Additionally, there was no difference in the relationship between vitamin D and HUA based on age stratification (age < 70, ≥70; Table 3, Supplementary Table S2). Sensitivity analyses, which excluded participants with CKD or included those taking vitamin D supplements, confirmed the robustness of these findings (Supplementary Tables S24–S40).

**Table 3:** Association between vitamin D and HUA.

**Sex**	**Vitamin D**					**P**		**Vitamin D**			**P**	
**Female**	**Continuous (*****n*** = **25,895)**	**Q1 (**<**16.8 ng/ml) (*****n*** = **6,449)**	**Q2 (16.8–20.8 ng/ml) (*****n*** = **6,463)**	**Q3 (20.8–24.6 ng/ml) (*****n*** = **6,471)**	**Q4 (**≥**24.6 ng/ml) (*****n*** = **6,451)**	***P*** **for trend**	***P*** **for FDR**	<**20 ng/ml (*****n*** = **11,576)**	**20–30 ng/ml (*****n*** = **12,537)**	≥**30 ng/ml (*****n*** = **1,721)**	***P*** **for trend**	***P*** **for FDR**
M1 [β (95% CI)]	1.01 (1.00, 1.01)	Ref	1.05 (0.95, 1.17)	1.09 (0.98, 1.20)	1.09 (0.98, 1.20)	0.091	0.196	Ref	1.04 (0.97, 1.12)	1.11 (0.95, 1.28)	0.134	0.294
M2 [β (95% CI)]	1.01 (1.00, 1.01)	Ref	1.07 (0.96, 1.19)	1.09 (0.98, 1.22)	1.12 (1.00, 1.25)	0.049	0.196	Ref	1.06 (0.98, 1.15)	1.12 (0.95, 1.31)	0.074	0.246
**Male**	**Continuous (*****n*** = **15,116)**	**Q1 (**<**22.4 ng/ml) (*****n*** = **3,775)**	**Q2 (22.4–26.2 ng/ml) (*****n*** = **3,776)**	**Q3 (26.2–30.1 ng/ml) (*****n*** = **3,772)**	**Q4 (**≥**30.1 ng/ml) (*****n*** = **3,778)**			<**20 ng/ml (*****n*** = **2,112)**	**20–30 ng/ml (*****n*** = **9,178)**	≥**30 ng/ml (*****n*** = **3,811)**		
M1 [β (95% CI)]	1.00 (0.99, 1.00)	Ref	0.96 (0.87, 1.06)	0.97 (0.87, 1.07)	0.94 (0.85, 1.04)	0.249	0.249	Ref	0.98 (0.88, 1.08)	0.94 (0.84, 1.06)	0.305	0.407
M2 [β (95% CI)]	1.00 (0.99, 1.00)	Ref	0.96 (0.86, 1.08)	0.99 (0.89, 1.11)	0.94 (0.84, 1.05)	0.281	0.281	Ref	0.98 (0.87, 1.10)	0.93 (0.82, 1.06)	0.249	0.332
**Male**<**70**	**Continuous (*****n*** = **9,368)**	**Q1 (**<**22 ng/ml) (*****n*** = **2,338)**	**Q2 (22–25.8 ng/ml) (*****n*** = **2,331)**	**Q3 (25.8–29.6 ng/ml) (*****n*** = **2,345)**	**Q4 (**≥**29.6 ng/ml) (*****n*** = **2,342)**			<**20 ng/ml (*****n*** = **1,413)**	**20–30 ng/ml (*****n*** = **5,748)**	≥**30 ng/ml (*****n*** = **2,195)**		
M1 [β (95% CI)]	1.00 (0.99, 1.00)	Ref	0.91 (0.80, 1.03)	0.91 (0.80, 1.03)	0.89 (0.79, 1.01)	0.098	0.196	Ref	0.94 (0.82, 1.06)	0.90 (0.77, 1.04)	0.147	0.294
M2 [β (95% CI)]	1.00 (0.99, 1.00)	Ref	0.90 (0.79, 1.04)	0.94 (0.82, 1.08)	0.90 (0.79, 1.04)	0.233	0.281	Ref	0.93 (0.81, 1.06)	0.88 (0.75, 1.03)	0.123	0.246
**Male** ≥**70**	**Continuous (*****n*** = **5,748)**	**Q1 (**<**22.99 ng/ml) (*****n*** = **1,437)**	**Q2 (22.99–26.76 ng/ml) (*****n*** = **1,435)**	**Q3 (26.76–30.70 ng/ml) (*****n*** = **1,437)**	**Q4 (**≥**30.70 ng/ml) (*****n*** = **1,436)**			<**20 ng/ml (*****n*** = **699)**	**20–30 ng/ml (*****n*** = **3,430)**	≥**30 ng/ml (*****n*** = **1,616)**		
M1 [β (95% CI)]	1.00 (1.00, 1.01)	Ref	0.97 (0.82, 1.15)	1.09 (0.92, 1.29)	1.09 (0.92, 1.28)	0.187	0.249	Ref	1.10 (0.91, 1.33)	1.10 (0.90, 1.36)	0.456	0.456
M2 [β (95% CI)]	1.01 (1.00, 1.02)	Ref	1.04 (0.86, 1.25)	1.14 (0.94, 1.37)	1.11 (0.92, 1.34)	0.180	0.281	Ref	1.16 (0.94, 1.44)	1.14 (0.90, 1.43)	0.460	0.460

M1: adjusted none; M2: adjusted age, sex, BMI, smoking, drinking, CREA, hyperlipidemia, HTN, and DM.

BMI, body mass index; UA, uric acid; CREA, creatinine; HTN, hypertension; DM, diabetes mellitus; HUA, hyperuricemia.

## 4 Discussion

Vitamin D deficiency (33.5%) and insufficiency (53%) were prevalent in this Chinese population, with men having a higher rate of insufficiency (60.8%) than women (48.5%). The relationship between vitamin D levels and serum UA varied by sex and age. In men under 70, an inverse J-shaped association was observed, with lower vitamin D linked to higher UA, attenuating at higher vitamin D levels. In women, a positive linear correlation between vitamin D and UA was seen, regardless of the variable type. Overall, the general population showed a pattern similar to women, indicating a broadly applicable positive linear relationship between vitamin D and UA. However, for HUA, while the general population exhibited an inverse J-shaped relationship, both genders displayed a positive linear trend, highlighting the differing effects of vitamin D on uric acid metabolism.

Several studies have explored the relationship between vitamin D and serum UA levels, revealing both complex and varied associations depending on population characteristics. Two meta-analyses (15, 33) found that vitamin D deficiency is linked to hyperuricemia. Similarly, an inverse U-shaped relationship was observed between vitamin D levels and serum UA, as well as the risk of elevated serum UA status, in a study involving 4,777 participants aged 6–18 years (*n* = 18,000) (21). Other research indicates a positive association between serum UA levels and 25-hydroxyvitamin D (25(OH)D), with the incidence of hyperuricemia increasing by 9.4% for every 10 nmol/L rise in 25(OH)D (*P* < 0.001) in a cohort of 9,220 subjects (17). A study in South Korea (10,864 participants) further emphasized a non-linear relationship between serum vitamin D and uric acid, showing a significant positive correlation within the vitamin D deficiency range (< 30 ng/ml) (34). In contrast, a cross-sectional study conducted in Zhejiang, China (7,086 participants), found an inverse U-shaped relationship between 25(OH)D and serum UA, identifying a threshold of 28.82 ng/ml, below which lower vitamin D levels were associated with an increased risk of hyperuricemia (OR: 1.0146, *P* = 0.0148), while higher levels were protective (OR: 0.9616, *P* = 0.0164) (20). Aadditionally, two studies involving adults aged 18 and older from the 2007–2014 National Health and Nutrition Examination Survey (NHANSE) in the United States found a significant negative association between vitamin D and HUA. Additionally, in the stratified analysis by gender, both studies found no gender differences in the relationship between vitamin D and UA/HUA (18, 19). These findings suggest that while the relationship between vitamin D and serum UA is multifaceted, it is influenced by both vitamin D status and the demographic characteristics of the studied populations. Besides, vitamin D and UA metabolism is regulated by genetic factors. It is worth noting that a single genetic variation site can explain 1%−4% of the variation in 25(OH)D levels across different populations (35–39). This variability underscores the need for further longitudinal and interventional research to establish the potential causal links and clinical implications of vitamin D and serum UA levels.

Over half of the Chinese participants in the present studies had vitamin D insufficiency (25(OH)D < 50 nmol/L). Vitamin D insufficiency/deficiency has been observed in various age groups across China. A meta-analysis by Zhang et al. (40) found that the prevalence of vitamin D deficiency (25(OH)D < 25 nmol/L) in Mainland China between 2000–2012 was approximately 30%. A cross-sectional study from the 2010 to 2013 China National Nutrition and Health Survey (CNNHS) reported that 12% of women and 7.8% of men over 60 had vitamin D deficiency (25(OH)D < 30 nmol/L), while 32% of women and 26.3% of men had insufficiency (25(OH)D < 50 nmol/L) (41). The 2010–2012 CNNHS also revealed that 7.2% of children and adolescents had vitamin D deficiency (25(OH)D < 25 nmol/L), and 42% had insufficiency (25(OH)D < 50 nmol/L) (42). These findings collectively highlight the significant burden of vitamin D deficiency and insufficiency in the general Chinese population.

The published evidence suggests that a minimum serum level of 25(OH)D is 30 ng/ml protects against less than one-third of common disorders, including musculoskeletal and calcium homeostasis. In contrast, when serum 25(OH)D levels range from 50 to 80 ng/ml, it can protect against 99% of health conditions without having any negative consequences (43). However, our results suggest that the theory of “the higher, the better” for vitamin D may not be entirely supported, vitamin D levels were inversely associated with UA in males under 70. but when vitamin D levels exceeded the above range, this inverse association disappeared, and serum UA increased with the increase of vitamin D level. The potential biological explanation for this finding is as follows: in men aged ≥70, declines in kidney function, vitamin D receptor (VDR) activity, and androgen levels reduce the bidirectional regulatory effects of vitamin D. In women, lower VDR expression due to vitamin D levels and estrogen influences, coupled with an increased risk of calcium metabolism disorders post-menopause, results in the harmful effects of high-dose vitamin D outweighing its “uric acid excre tion-promoting” benefits. Consequently, the relationship between vitamin D levels and the outcome does not exhibit a reverse J-shaped curve (15). Similarly, in the females of our study, higher vitamin D levels do not necessarily correlate with lower UA levels. When vitamin D exceeds a certain threshold, it can lead to HUA, which is associated with a variety of diseases. It is important to note that the phrase “the lower the serum UA, the better” is outdated. Considering that vitamin D and serum UA are positively correlated in females and males older than 70 years old in our study, too low and too high vitamin D levels may be associated with hypouricemia and HUA, respectively. Therefore vitamin D levels should be maintained within an optimal range. Additionally, our study found an L-shaped relationship between vitamin D and serum UA in males under 70 years. This emphasizes the importance of considering optimal vitamin D levels across different age groups and sexes.

Although there is some evidence of a relationship between serum vitamin D and serum UA, the causal relationship remains unclear. It has been reported that 25(OH)D is metabolized by the enzyme 1α-hydroxylase in the kidneys into 1,25-dihydroxy (1,25(OH)_2_D). This active form of vitamin D inhibits parathyroid hormone (PTH) synthesis, both directly by activating the vitamin D receptor in the parathyroid glands, and indirectly by stimulating intestinal calcium absorption, which leads to a transient increase in serum ionized calcium level (44). Furthermore, PTH downregulates the urate exporter, ATP-binding cassette transporter G2 transporter (ABCG2), which leads to decreased urinary UA excretion and subsequent accumulation of serum UA (45). Elevated UA inhibits the expression of the CYP27B1 gene, which encodes the enzyme 1α-hydroxylase. This enzyme is responsible for converting 25(OH)D into 1,25(OH)_2_D in the renal proximal tubule, resulting in increased level of 25(OH)D (46, 47). Despite the proposed interaction mechanism between vitamin D, PTH, and UA, the causal relationship between vitamin D and serum UA remains controversial. Two bidirectional analyses investigating this relationship reported conflicting results. Han et al. (48) suggested a causal association of genetically predicted UA on 25(OH)D. Specifically, they found that each 1 mg/dl increase in UA was associated with a decrease of 0.74 nmol/L of 25(OH)D. However, no causal relationship was observed between 25(OH)D and serum UA. Another bidirectional analysis conducted by Thakkinstian et al. constructed two causal pathways: rs2282679 → 25(OH)D → UA and rs2231142 → UA → 25(OH). They found each minor C allele in rs2282679 led to a decreasein 25(OH)D and then significantly decreased the UA by 0.0236 unit. For the second pathway, they found each T allele copy for rs2231142 increased UA levels, and subsequently increasing 25(OH)D by 0.0806 unit. In addition, this study also demonstrated a positive correlation between vitamin D levels and serum UA, which is partly consistent with our results (49). These findings suggest that clarifying the causal relationship between vitamin D and UA requires further validation through longitudinal cohort studies and interventional research. This is particularly important considering confounding factors such as impaired renal function, which may lead to both elevated UA levels and altered vitamin D metabolism, as well as the potential for reverse causality.

To date, no real-world population survey with such a large sample size across a broad age range has assess vitamin D levels in the Chinese population, the only existing studies include a 2013 meta-analysis (34) and several smaller studies focused on specific age groups, such as adolescents (42) or older adults (35). Furthermore, all participants in our study were included during the same season, which helps reduce bias, as vitamin D levels can be influenced by sunlight exposure. Additionally, the methods of vitamin D detection in the existing studies were inaccurate, either as a crude assessment using dietary questionnaires or chemiluminescence method (13, 16, 17), rather than applying the gold standard for vitamin D detection: LC-MS/MS. we used LC-MS, the gold standard for vitamin D detection, ensuring the accuracy of our measurements. To account for individual variations we also assessed the relationship between UA and vitamin D across gender and age groups.

This study has several limitations. Firstly, due to its cross-sectional design, it is not possible to establish a causal relationship between vitamin D levels and serum UA. Additionally, residual confounding may have affected the results, as other unmeasured factors—such as lifestyle, physical activity, or underlying health conditions—could have influenced both vitamin D levels and serum UA. Moreover, the study lacked data on dietary intake or sunlight exposure, both of which are important determinants of vitamin D status. Although this study provides valuable insights into the association between vitamin D levels and serum UA, the findings are not sufficient to directly inform individualized vitamin D recommendations. Further research, incorporating longitudinal data and considering dietary and sunlight exposure factors, is needed to develop personalized guidelines for vitamin D supplementation in the Chinese population.

In conclusion, our large-scale study suggests a significant vitamin D deficiency/insufficiency in the Chinese population. A sex- and age-specific association between vitamin D levels and both serum UA and HUA was observed in middle-aged to elderly Chinese individuals. If this potential causal relationship is further confirmed through longitudinal studies, our findings could pave the way for new strategies in the prevention and treatment of HUA, positioning vitamin D as a promising therapeutic target.

## Data Availability

The raw data supporting the conclusions of this article will be made available by the authors, without undue reservation.

## Supplementary material

Supplementary Table S1 Association between vitamin D and serum UA in the general population.

Supplementary Table S2 Association between vitamin D and HUA in the general population.

Supplementary Table S3 Association between vitamin D and serum UA.

Supplementary Table S4 Association between vitamin D and HUA.

Supplementary Table S5 VIF and metric.

Supplementary Tables S6–S14 Sensitivity analysis of the relationship between vitamin D and serum UA in non-CKD Subjects.

Supplementary Tables S15–S23 Sensitivity analysis of the association between vitamin D and serum UA including vitamin D intake.

Supplementary Tables S24–31 Sensitivity analysis of the relationship between vitamin D and HUA in non-CKD subjects.

Supplementary Tables S32–S40 Sensitivity analysis of the association between vitamin D and HUA including vitamin D intake.

## References

[1] Otani N Ouchi M Mizuta E Morita A Fujita T Anzai N . Dysuricemia-a new concept encompassing hyperuricemia and hypouricemia. Biomedicines. (2023) 11:1255. doi: 10.3390/biomedicines11051255 37238926 PMC10215565

[2] Wang S Shu Z Tao Q Yu C Zhan S Li L. Uric acid and incident chronic kidney disease in a large health check-up population in Taiwan. Nephrology. (2011) 16:767–76. doi: 10.1111/j.1440-1797.2011.01513.x 21854506

[3] Tana C Ticinesi A Prati B Nouvenne A Meschi T. Uric acid and cognitive function in older individuals. Nutrients. (2018) 10:975. doi: 10.3390/nu10080975 30060474 PMC6115794

[4] Haig A. On uric acid and arterial tension. Br Med J. (1889) 1:288–91. doi: 10.1136/bmj.1.1467.288 20752582 PMC2154674

[5] Giustina A Bilezikian JP Adler RA Banfi G Bikle DD Binkley NC . Consensus statement on vitamin D status assessment and supplementation: whys, whens, and hows. Endocr Rev. (2024) 45:625–54. doi: 10.1210/endrev/bnae009 38676447 PMC11405507

[6] de la Guía-Galipienso F Martínez-Ferran M Vallecillo N Lavie CJ Sanchis-Gomar F Pareja-Galeano H. Vitamin D and cardiovascular health. Clin Nutr. (2021) 40:2946–57. doi: 10.1016/j.clnu.2020.12.025 33397599 PMC7770490

[7] Carlberg C Velleuer E. Vitamin D and the risk for cancer: a molecular analysis. Biochem Pharmacol. (2022) 196:114735. doi: 10.1016/j.bcp.2021.114735 34411566

[8] Szymczak-Pajor I Drzewoski J Sliwińska A. The molecular mechanisms by which vitamin D prevents insulin resistance and associated disorders. Int J Mol Sci. (2020) 21:6644. doi: 10.3390/ijms21186644 32932777 PMC7554927

[9] Athanassiou L Kostoglou-Athanassiou I Koutsilieris M Shoenfeld Y. Vitamin D and autoimmune rheumatic diseases. Biomolecules. (2023) 13:709. doi: 10.3390/biom13040709 37189455 PMC10135889

[10] Latic N Erben RG. Vitamin D and cardiovascular disease, with emphasis on hypertension, atherosclerosis, and heart failure. Int J Mol Sci. (2020) 21:6483. doi: 10.3390/ijms21186483 32899880 PMC7555466

[11] Pludowski P Takacs I Boyanov M Belaya Z Diaconu CC Mokhort T . Clinical practice in the prevention, diagnosis and treatment of vitamin D deficiency: a Central and Eastern European expert consensus statement. Nutrients. (2022) 14:1483. doi: 10.3390/nu14071483 35406098 PMC9002638

[12] Cashman KD. Global differences in vitamin D status and dietary intake: a review of the data. Endocr Connect. (2022) 11:e210282. doi: 10.1530/EC-21-0282 34860171 PMC8789021

[13] Chien KL Hsu HC Chen PC Lin HJ Su TC Chen MF . Total 25-hydroxyvitamin D concentration as a predictor for all-cause death and cardiovascular event risk among ethnic Chinese adults: a cohort study in a Taiwan community. PLoS ONE. (2015) 10:e0123097. doi: 10.1371/journal.pone.0123097 25807387 PMC4373875

[14] Radulović Ž Zupan ZP Tomazini A Varda NM. Vitamin D in pediatric patients with obesity and arterial hypertension. Sci Rep. (2021) 11:19591. doi: 10.1038/s41598-021-98993-8 34599252 PMC8486804

[15] Isnuwardana R Bijukchhe S Thadanipon K Ingsathit A Thakkinstian A. Association between vitamin D and uric acid in adults: a systematic review and meta-analysis. Horm Metab Res. (2020) 52:732–41. doi: 10.1055/a-1240-5850 33049785 PMC7556437

[16] Chan HL Elkhashab M Trinh H Tak WY Ma X Chuang WL . Association of baseline vitamin D levels with clinical parameters and treatment outcomes in chronic hepatitis B. J Hepatol. (2015) 63:1086–92. doi: 10.1016/j.jhep.2015.06.025 26143444

[17] Chen Y Cheng J Chen Y Wang N Xia F Chen C . Association between serum vitamin D and uric acid in the eastern Chinese population: a population-based cross-sectional study. BMC Endocr Disord. (2020) 20:79. doi: 10.1186/s12902-020-00560-1 32493273 PMC7268462

[18] Han Y Han K Zhang Y Zeng X. Serum 25-hydroxyvitamin D might be negatively associated with hyperuricemia in U.S. adults: an analysis of the National Health and Nutrition Examination Survey 2007–2014. J Endocrinol Invest. (2022) 45:719–29. doi: 10.1007/s40618-021-01637-x 34435335 PMC8918159

[19] Zhang YY Qiu HB Tian JW. Association between vitamin D and hyperuricemia among adults in the United States. Front Nutr. (2020) 7:592777. doi: 10.3389/fnut.2020.592777 33330592 PMC7714933

[20] Li ST Wang YL Ni FH Sun T. Association between 25 hydroxyvitamin D and serum uric acid level in the Chinese general population: a cross-sectional study. BMC Endocr Disord. (2024) 24:187. doi: 10.1186/s12902-024-01723-0 39261907 PMC11391835

[21] Ma Z Xiong T Li Y Kong B Lu W Zhang Z . The inverted U-shaped association between serum vitamin D and serum uric acid status in children and adolescents: a large cross-sectional and longitudinal analysis. Nutrients. (2024) 16:1492. doi: 10.3390/nu16101492 38794730 PMC11124299

[22] Liu ZY Liu S Yao X Wang CY Song Y Bi YM . A cohort study of serum 25-hydroxyvitamin D levels and the risk of hyperlipidaemia in adults. Front Nutr. (2025) 11:1492621. doi: 10.3389/fnut.2024.1492621 39925969 PMC11802281

[23] Xu BP Shi H. Precision nutrition: concept, evolution, and future vision. Precis Nutr. (2022) 1:e00002. doi: 10.1097/PN9.0000000000000002

[24] Song Y Chen P Zalloua PA Li J Shi H. Precision nutrition: 8 stages and 5 dimensions. Precis Nutr. (2023) 2:e00057. doi: 10.1097/PN9.0000000000000057

[25] Shi H Li J Mao C Wang B Qin X Bao H . China precision nutrition and health-KAP real world study (CPN-KAPS): rationale, study design, and protocol. Precis Nutr. (2022) 1:e00021. doi: 10.1097/PN9.0000000000000021

[26] Li J Mao C Xu B Zhang Y Wang B Tang G . China precision nutrition and health-KAP (knowledge, attitude, and practice) real-world study (CPNAS): enrollment progress and baseline characteristics. Precis Nutr. (2025) 4:e00095. doi: 10.1097/PN9.0000000000000095

[27] Chen P Lin G Lin L Song Y Huan J Tian M. Simultaneous determination of vitamins A, D, E, K in human plasma. Precis Nutr. (2024) 3:e00086. doi: 10.1097/PN9.0000000000000086

[28] Holick MF Binkley NC Bischoff-Ferrari HA Gordon CM Hanley DA Heaney RP . Evaluation, treatment, and prevention of vitamin D deficiency: an endocrine society clinical practice guideline. J Clin Endocrinol Metab. (2011) 96:1911–30. doi: 10.1210/jc.2011-0385 21646368

[29] Fang HQ Jiang P Wang YJ Fan WC Qu PF Li N . Dietary guidelines for adult hyperuricemia and gout (2024 Edition). J Hyg Res. (2024) 53:352–6. Chinese. doi: 10.19813/j.cnki.weishengyanjiu.2024.03.002

[30] Williams B Mancia G Spiering W Agabiti Rosei E Azizi M Burnier M . 2018 ESC/ESH Guidelines for the management of arterial hypertension. Eur Heart J. (2018) 39:3021–104. doi: 10.1093/eurheartj/ehy339 30165516

[31] American Diabetes Association Professional Practice Committee. 2. Diagnosis and classification of diabetes: standards of care in diabetes-−2024. Diabetes Care. (2024) 47:S20–42. doi: 10.2337/dc24-S002 38078589 PMC10725812

[32] Lu Y Zhang H Lu J Ding Q Li X Wang X . Prevalence of dyslipidemia and availability of lipid-lowering medications among primary health care settings in China. JAMA Netw Open. (2021) 4:e2127573. doi: 10.1001/jamanetworkopen.2021.27573 34586366 PMC8482054

[33] Charoenngam N Ponvilawan B Ungprasert P. Vitamin D insufficiency and deficiency are associated with a higher level of serum uric acid: a systematic review and meta-analysis. Mod Rheumatol. (2020) 30:385–90. doi: 10.1080/14397595.2019.1575000 30689484

[34] Lee HR Joo NS. Non-linear associations between serum vitamin D and uric acid in Korean adults: 2022–2023 KNHANES data. Nutrients. (2025) 17:2398. doi: 10.3390/nu17152398 40805984 PMC12348428

[35] Wang TJ Zhang F Richards JB Kestenbaum B van Meurs JB Berry D . Common genetic determinants of vitamin D insufficiency: a genome-wide association study. Lancet. (2010) 376:180–8. doi: 10.1016/S0140-6736(10)60588-0 20541252 PMC3086761

[36] Nissen J Vogel U Ravn-Haren G Andersen EW Madsen KH Nexø BA . Common variants in CYP2R1 and GC genes are both determinants of serum 25-hydroxyvitamin D concentrations after UVB irradiation and after consumption of vitamin D_3_-fortified bread and milk during winter in Denmark. Am J Clin Nutr. (2015) 101:218–27. doi: 10.3945/ajcn.114.092148 25527766

[37] Ordóñez-Mena JM Maalmi H Schöttker B Saum KU Holleczek B Wang TJ . Genetic variants in the vitamin D pathway, 25(OH)D levels, and mortality in a large population-based cohort study. J Clin Endocrinol Metab. (2017) 102:470–7. doi: 10.1210/jc.2016-2468 27732326

[38] Zhang Z He JW Fu WZ Zhang CQ Zhang ZL. An analysis of the association between the vitamin D pathway and serum 25-hydroxyvitamin D levels in a healthy Chinese population. J Bone Miner Res. (2013) 28:1784–92. doi: 10.1002/jbmr.1926 23505139

[39] Li SS Gao LH Zhang XY He JW Fu WZ Liu YJ . Genetically low vitamin D levels, bone mineral density, and bone metabolism markers: a Mendelian randomisation study. Sci Rep. (2016) 6:33202. doi: 10.1038/srep33202 27625044 PMC5021966

[40] Zhang W Stoecklin E Eggersdorfer M. A glimpse of vitamin D status in Mainland China. Nutrition. (2013) 29:953–7. doi: 10.1016/j.nut.2013.01.010 23594582

[41] Chen J Yun C He Y Piao J Yang L Yang X. Vitamin D status among the elderly Chinese population: a cross-sectional analysis of the 2010–2013 China National Nutrition and Health Survey (CNNHS). Nutr J. (2017) 16:3. doi: 10.1186/s12937-016-0224-3 28088200 PMC5237548

[42] Hu Y Chen J Wang R Li M Yun C Li W . Vitamin D nutritional status and its related factors for Chinese children and adolescents in 2010–2012. Nutrients. (2017) 9:1024. doi: 10.3390/nu9091024 28914773 PMC5622784

[43] Wimalawansa SJ. Infections and autoimmunity-the immune system and vitamin D: a systematic review. Nutrients. (2023) 15:3842. doi: 10.3390/nu15173842 37686873 PMC10490553

[44] Ponvilawan B Charoenngam N. Vitamin D and uric acid: is parathyroid hormone the missing link? J Clin Transl Endocrinol. (2021) 25:100263. doi: 10.1016/j.jcte.2021.100263 34307053 PMC8283022

[45] Sugimoto R Watanabe H Ikegami K Enoki Y Imafuku T Sakaguchi Y . Down-regulation of ABCG2, a urate exporter, by parathyroid hormone enhances urate accumulation in secondary hyperparathyroidism. Kidney Int. (2017) 91:658–70. doi: 10.1016/j.kint.2016.09.041 27988213

[46] Chen W Roncal-Jimenez C Lanaspa M Gerard S Chonchol M Johnson RJ . Uric acid suppresses 1 alpha hydroxylase *in vitro* and *in vivo*. Metabolism. (2014) 63:150–60. doi: 10.1016/j.metabol.2013.09.018 24269076 PMC3859721

[47] Ebert R Schütze N Adamski J Jakob F. Vitamin D signaling is modulated on multiple levels in health and disease. Mol Cell Endocrinol. (2006) 248:149–59. doi: 10.1016/j.mce.2005.11.039 16406653

[48] Han Y Zhang Y Zeng X. Assessment of causal associations between uric acid and 25-hydroxyvitamin D levels. Front Endocrinol. (2022) 13:1024675. doi: 10.3389/fendo.2022.1024675 36583002 PMC9792848

[49] Thakkinstian A Anothaisintawee T Chailurkit L Ratanachaiwong W Yamwong S Sritara P . Potential causal associations between vitamin D and uric acid: bidirectional mediation analysis. Sci Rep. (2015) 5:14528. doi: 10.1038/srep14528 26417870 PMC4586492

